# PD173 Semi-Automation Of Systematic Literature Review Title And Abstract Screening Using Text Mining And Classification Techniques

**DOI:** 10.1017/S0266462324003994

**Published:** 2025-01-07

**Authors:** Ciara Morgan, Niamh Carey, Arthur White

## Abstract

**Introduction:**

Researchers are increasingly faced with the challenge of producing a robust systematic literature review (SLR) within the confines of time and budget. Semi-automating the title and abstract screening phase has been proposed to reduce the workload burden of SLRs. This research aimed to evaluate the efficacy of text mining and machine learning techniques in the semi-automation of the title and abstract screening phase of SLRs.

**Methods:**

Two SLRs that had been manually screened by one screener (manual SLRs) were examined. The titles and abstracts of these SLRs were tokenized and the datasets were split into training and test sets. Support vector machines (SVM), Naïve Bayes (NB), and k-nearest neighbors (k-NN) classification machine learning models were used to predict whether documents in the test set were classed as relevant during the manual SLR. Diagnostic evaluation was carried out using Shapley Additive explanations and local interpretable model-agnostic explanations to explain the predictions of the optimal model.

**Results:**

SVM achieved a sensitivity of one for both SLRs, successfully identifying all documents classed as relevant in the manual SLR. For one SLR, diagnostic evaluation indicated that the model used relevant features to generate its predictions. For the second SLR, the model had the tendency to predict using less relevant or misinterpreted variables. This may be because certain features (i.e., words) the model was trained on had different meanings depending on the clinical context and were present in both relevant and irrelevant citations. This demonstrates the inability of such models to extract semantic meaning from text.

**Conclusions:**

For the second SLR, domain expertise was required to evaluate the features driving the SVM model predictions. This highlights the importance of using discretion when determining the trustworthiness of results generated by such models. This is important to researchers, who need assurance that the use of such techniques will not compromise the validity of their results.

